# Sex-specific differences in basilar artery vasospasm after subarachnoid hemorrhage: evidence from a rabbit model

**DOI:** 10.3389/fneur.2026.1739644

**Published:** 2026-06-03

**Authors:** İskender Samet Daltaban, Ayhan Kanat, Mehmet Selim Gel, Mehmet Dumlu Aydin, Rabia Demirtas, Songül Turğut

**Affiliations:** 1 Department of Neurosurgery, A Life Hospital, Ankara, Türkiye; 2 Department of Neurosurgery, Recep Tayyip Erdogan University, Rize, Türkiye; 3 Department of Neurosurgery, Kanuni Research and Training Hospital, Trabzon, Türkiye; 4 Department of Neurosurgery, Ataturk University, Erzurum, Türkiye; 5 Department of Pathology, Ataturk University, Erzurum, Türkiye; 6 Medicana International Ankara Hospital, Department of Neurology, Ankara, Türkiye

**Keywords:** subarachnoid hemorrhage, vasospasm, intracranial, basilar artery, estradiol, sex differences, rabbits

## Abstract

**Background:**

Subarachnoid hemorrhage (SAH) remains a devastating cerebrovascular disorder in which cerebral vasospasm is a key determinant of secondary injury and outcome. Sex differences are well-documented clinically, women exhibit a higher incidence of aneurysmal SAH and often worse outcomes, but whether biological sex influences vasospasm severity in controlled preclinical settings remains unclear.

**Methods:**

In a randomized, controlled rabbit model, we investigated sex-specific differences in basilar artery (BA) vasospasm after SAH. Twenty-four New Zealand White rabbits (12 male, 12 female) were allocated to Control, SHAM, or SAH groups. SAH was induced by cisterna magna injection of autologous blood; SHAM animals received saline. On Day 7, BA segments were harvested for quantitative assessment of vasospasm using the Vasospasm Index (VSI: wall area/lumen area) and blinded qualitative histopathological scoring of smooth muscle contraction, endothelial integrity, internal elastic membrane configuration, and adventitial thickness. Nonparametric statistical tests were used, with male vs. female comparison in SAH as the primary endpoint.

**Results:**

Control and SHAM animals showed no significant sex differences in VSI (Control: *p* = 0.47; SHAM: *p* = 0.06). In contrast, SAH induced severe vasospasm in males (VSI 2.34 ± 0.63) compared with moderate vasospasm in females (1.27 ± 0.49, *p* < 0.001). Histopathology corroborated these findings, with severe endothelial disruption and smooth muscle hypercontraction in males versus moderate alterations in females.

**Conclusion:**

Male rabbits exhibited significantly more severe vasospasm than females after SAH, despite human epidemiology suggesting greater vulnerability in women. These results demonstrate sex as a fundamental biological variable in SAH pathophysiology and underscore the need for mechanistic studies exploring hormonal, autonomic, and inflammatory mediators. Incorporating sex into experimental design may ultimately inform sex-specific therapeutic strategies for SAH.

## Introduction

Subarachnoid hemorrhage (SAH), particularly aneurysmal SAH (aSAH), is recognized as one of the most devastating forms of stroke, associated with high early mortality and long-term disability in survivors (1, 2). Despite advances in neurosurgical techniques and critical care, the prognosis after aSAH, especially when complicated by cerebral vasospasm, remains poor (3). While clinical studies in humans frequently emphasize functional outcomes [e.g., delayed cerebral ischemia (DCI), infarction, hydrocephalus], the studies focuses on anatomical vasospasm of the basilar artery as a mechanistic endpoint are lacking. Accordingly, all interpretations are made with this endpoint in mind and not as direct surrogates for clinical disability or DCI.

SAH also demonstrates a clear sex-specific epidemiology: women account for a significantly greater proportion of aSAH cases compared with men, with approximately 65% of patients being female and exhibiting a 1.3-fold higher relative risk (4). Furthermore, several clinical studies have shown that female patients tend to experience worse outcomes, including higher rates of DCI and hydrocephalus, even after adjusting for potential confounders such as age and treatment modality (5, 6).

These observations have directed increasing attention toward the role of sex hormones and other biological sex differences in cerebrovascular pathology. Estrogen, particularly 17β-estradiol, exerts vasoprotective effects by enhancing endothelial nitric oxide synthase (eNOS) activity, promoting nitric oxide–mediated vasodilation, and suppressing proinflammatory cytokine expression, thereby improving vascular resilience to injury and spasm (7, 8). Experimental animal studies corroborate these findings: bilateral oophorectomy, which induces estrogen deficiency, significantly accelerates the formation and enlargement of intracranial aneurysms in rats, whereas hormone replacement mitigates these effects (9, 10). Additional evidence confirms that estrogen deficiency exacerbates cerebrovascular pathology, while estrogen supplementation reduces cerebral artery tone and improves vasoreactivity (11). Collectively, these data suggest that biological sex, through hormonal and vascular mechanisms, fundamentally modulates the cerebrovascular response to hemorrhagic insults such as SAH.

Given the contrasting epidemiological evidence in humans and the paucity of experimental data, the present study was designed to investigate whether biological sex influences the severity of basilar artery vasospasm following SAH in a rabbit model. By quantitatively and histopathologically assessing vasospasm in male and female animals, we sought to clarify the extent to which sex-specific factors contribute to cerebrovascular responses after hemorrhagic stroke. Because our primary endpoint is arterial narrowing/histopathology rather than clinical DCI, we interpret any sex differences in the context of vascular biology and explicitly caution against direct extrapolation to patient outcomes without further translational work.

## Methods

### Study design and oversight

This was a randomized, controlled, parallel-group animal study with a 2 (sex: male/female) × 3 (condition: Control/SHAM/SAH) factorial structure. The primary endpoint was basilar artery (BA) vasospasm severity at Day 7, quantified by the Vasospasm Index (VSI). Secondary endpoints were predefined qualitative histopathological features of vasospasm (media smooth muscle contraction, endothelial integrity, internal elastic membrane configuration, and adventitial thickness). All procedures complied with the National Institutes of Health Guide for the Care and Use of Laboratory Animals and were approved by the Atatürk University Animal Research Ethics Committee (Approval No. 2021-252).

### Animals and housing

Twenty-four adult (3-year-old) New Zealand White rabbits were used (12 male, 12 female). Animals were acclimatized ≥7 days before experimentation and maintained under standard conditions (12:12 h light–dark cycle; temperature- and humidity-controlled facility) with ad libitum access to food and water. Health status was verified by a veterinarian prior to enrollment. Female rabbits were intact (not ovariectomized) and were not subjected to exogenous hormone manipulation. Female estrous staging was not performed and serum sex hormones were not measured; potential cycle-related variability is addressed in the Limitations and proposed future work.

### Group allocation, randomization, and blinding

Within each sex, animals were randomly assigned (computer-generated random sequence) to Control (*n* = 3), SHAM (*n* = 4), or SAH (*n* = 5) groups. Allocation was performed by a technician not involved in outcome assessment. Investigators performing histopathological measurements and qualitative ratings were blinded to sex and group.

### Anesthesia, peri-procedural monitoring, and humane endpoints

General anesthesia was induced by intramuscular injection of ketamine (25 mg/kg), lidocaine (15 mg/kg), and acepromazine (1 mg/kg). Depth of anesthesia was confirmed by the absence of pedal and corneal reflexes before any surgical procedure. Animals were kept normothermic throughout and continuously monitored for respiration and heart rate. Prespecified humane endpoints (e.g., unremitting respiratory distress, refractory seizures, or moribund state) mandated immediate euthanasia. After surgery, all animals received identical supportive care and daily monitoring for 7 days (neurologic status, activity, and food/water intake).

### Model induction

#### SAH induction

A single-injection cisterna magna model was selected to induce a global SAH sufficient to elicit day-7 vasospasm while minimizing repeated manipulation. Blood was infused slowly over ~60 s to limit abrupt ICP spikes; intracranial pressure was not invasively monitored, which we acknowledge as a limitation, but all procedures used an identical injection rate and head position to standardize the stimulus across animals. One mL of autologous arterial blood (central ear artery) was slowly injected into the cisterna magna under sterile conditions using a 22-gauge needle with the head flexed to open the cisterna. The needle was withdrawn carefully to minimize CSF leakage.

#### SHAM procedure

SHAM animals underwent the identical procedure with 1 mL sterile 0.9% saline.

#### Control

Control animals underwent no cisternal injection.

### Tissue collection, perfusion-fixation, and processing

On Day 7, animals were deeply anesthetized using the same protocol (ketamine 25 mg/kg, lidocaine 15 mg/kg, acepromazine 1 mg/kg, intramuscularly) and then euthanized by transcardial perfusion under deep anesthesia, in strict accordance with the American Veterinary Medical Association (AVMA) Guidelines for the Euthanasia of Animals (2020). Adequate anesthesia depth was reconfirmed prior to euthanasia. Transcardial perfusion was then performed with heparinized saline followed by 10% buffered formalin to preserve vascular patency and prevent artifactual narrowing. Brains were immediately removed, and the basilar artery (BA) was dissected for post-fixation and histological processing as described below.

### Quantification of vasospasm (primary endpoint)

For each animal, digital micrographs were acquired under identical magnification with a calibrated microscope-camera system. At each of the 20 levels, the inner radius (*r*, lumen to endothelium) and outer radius (*R*, center to outer adventitia) were measured (three replicate tracings per section; averaged). Per animal, mean lumen radius (*r̄*) and mean outer radius (*R̄*) were computed and used to derive lumen area (*π r̄*^2^) and total vessel area (*π R̄*^2^). The Vasospasm Index (VSI) was defined as:


$$\begin{array}{l} \mathrm{VSI}=(\text{Vessel wall area})/(\text{Lumen area})\\ =(\pi {\bar{R}}^{2}-\pi {\bar{r}}^{2})/(\pi {\bar{r}}^{2})=({\bar{R}}^{2}-{\bar{r}}^{2})/{\bar{r}}^{2} \end{array}$$

By construction, higher VSI indicates more severe vasospasm (thicker wall and/or smaller lumen). For interpretability, we categorized VSI as mild (~1.0–1.5), moderate (1.5–2.0), and severe (>2.0).

### Qualitative histopathology (secondary endpoints)

A board-certified neuropathologist (blinded) assessed four canonical features of vasospasm in H&E sections:

(1) SM; contraction in the media; (2) E; integrity/deformation; (3) IEM; configuration (undulating vs. convoluted); (4) A; thickness/inflammation. Each was scored on a 4-point ordinal scale (none, mild, moderate, severe) using representative sections from each level; consensus was reached in case of ambiguity. Findings were summarized per animal and per group.

### Data quality and reproducibility safeguards

All measurements were performed in triplicate and averaged to reduce random error. Calibration of the optical system was verified before each session using a stage micrometer. Measurement order was randomized to mitigate drift. The analysis pipeline and raw measurement spreadsheets are available from the corresponding author upon reasonable request.

### Attrition and analysis population

Two rabbits (both assigned to SAH; one male, one female) died within 24 h of the injection due to anesthesia-related cardiorespiratory complications and were replaced to preserve planned group sizes. No other losses occurred. Analyses included all animals with complete Day-7 data; no imputation was applied.

### Statistical analysis

Analyses were performed in IBM SPSS Statistics v23. Continuous data are reported as mean ± SD; ordinal scores as median [IQR] where applicable. Given small per-group samples and non-normality concerns, we used Kruskal–Wallis tests for overall group differences, followed by Mann–Whitney *U* tests for prespecified pairwise comparisons. The primary comparison was male vs. female within the SAH group (VSI). Secondary comparisons included sex differences within SHAM and Control, and condition effects within each sex. Two-tailed *p* < 0.05 denoted statistical significance. A post-hoc power analysis, based on the observed VSI effect size between male-SAH and female-SAH groups, indicated ~85% power at *α* = 0.05. We prespecified a restricted set of pairwise contrasts (male vs. female within each condition; condition effects within each sex). Given this limited, hypothesis-driven set, we did not adjust for multiplicity; results are interpreted alongside effect sizes (rank-biserial correlation for Mann–Whitney, *η*^2^ for Kruskal–Wallis) and exact two-tailed *p* values. To acknowledge statistical multiplicity, we now clarify that all secondary comparisons are exploratory; importantly, the prespecified primary comparison (male vs. female VSI within SAH) remains highly significant (*p* < 0.001) and would remain significant under a conservative Bonferroni correction across the three sex comparisons (*α* = 0.0167).

## Results

### Animal outcomes and clinical observations

Of the 24 rabbits enrolled, 22 survived the full 7-day observation period. Two (one male, one female, both in the SAH group) died within 24 h due to anesthesia-related cardiorespiratory complications and were replaced to maintain group size. No other mortality occurred. SAH animals of both sexes exhibited typical post-hemorrhagic signs, including nuchal rigidity, lethargy, and transient loss of consciousness. Seizures, respiratory irregularities, and fever (>39 °C) were observed in several SAH animals but not in Controls. SHAM animals displayed mild, transient lethargy or neck stiffness during the first 48 h.

### Gross pathology

At necropsy, brains from SAH groups revealed diffuse subarachnoid clotting and leptomeningeal thickening, most prominently at the basal cisterns. The basilar artery (BA) in SAH animals appeared elongated, tortuous, and occasionally dolichoectatic. Grossly, BAs from Controls and SHAMs appeared normal, with smooth contours and no surface hemorrhage.

### Histopathology

**Figure 1:**
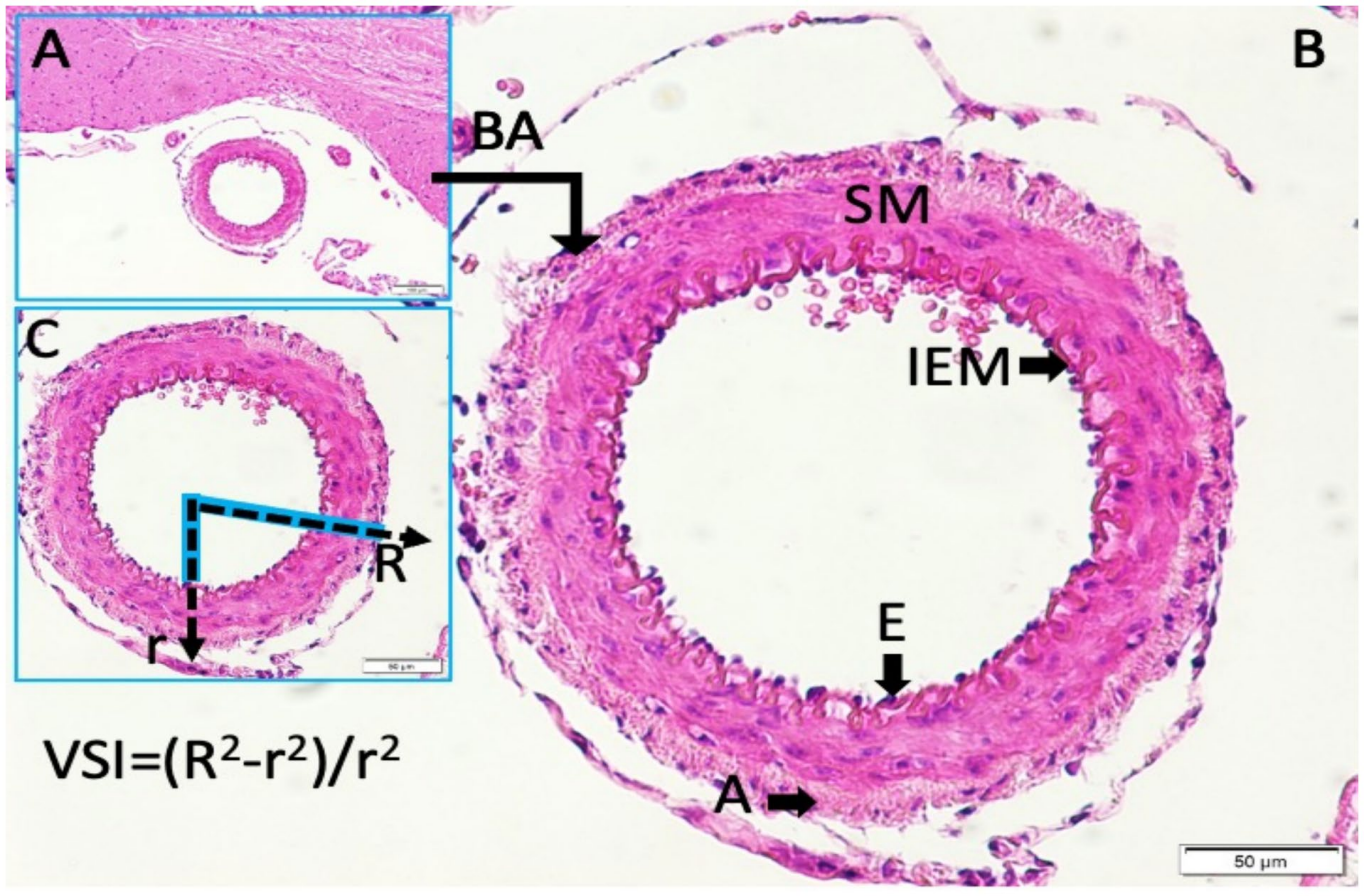
Histopathological appearances of basilar artery (BA) (LM, H&E, ×10/A); cellulary units of BA’s as smooth muscles (SM), endothelial cells (E), inner elastic membrane (IEM) and adventitia (A) (LM, H&E, 40/B) and VSI estimation method (C) is seen in a normal male rabbit.

**Figure 2:**
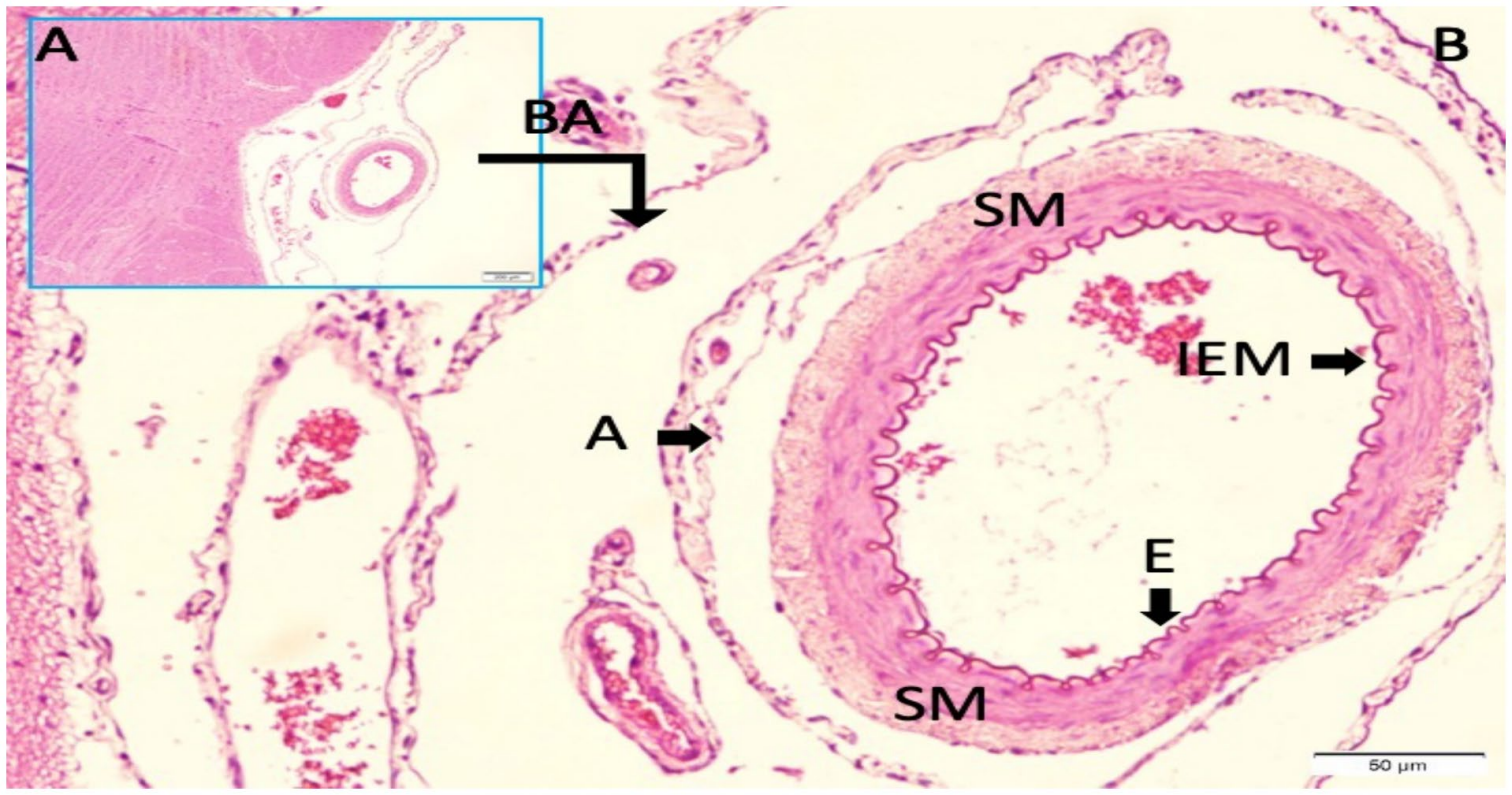
Histopathological appearances of basilar artery (BA) (LM, H&E, ×10/A); cellulary units of BA’s as smooth muscles (SM), endothelial cells (E), inner elastic membrane (IEM) and adventitia (A) (LM, H&E, 40/B) is seen in a normal female rabbit.

Control BAs showed intact architecture with organized smooth muscle (SM), a continuous endothelium (E), an undulating internal elastic membrane (IEM), and a thin adventitia (A). Representative micrographs of a normal male BA and female BA are shown in Figures 1, 2, respectively. These panels also present the cellular units (SM, E, IEM, A) at higher magnification; in Figure 1C, the VSI estimation method is schematized in the normal male specimen.

**Figure 3:**
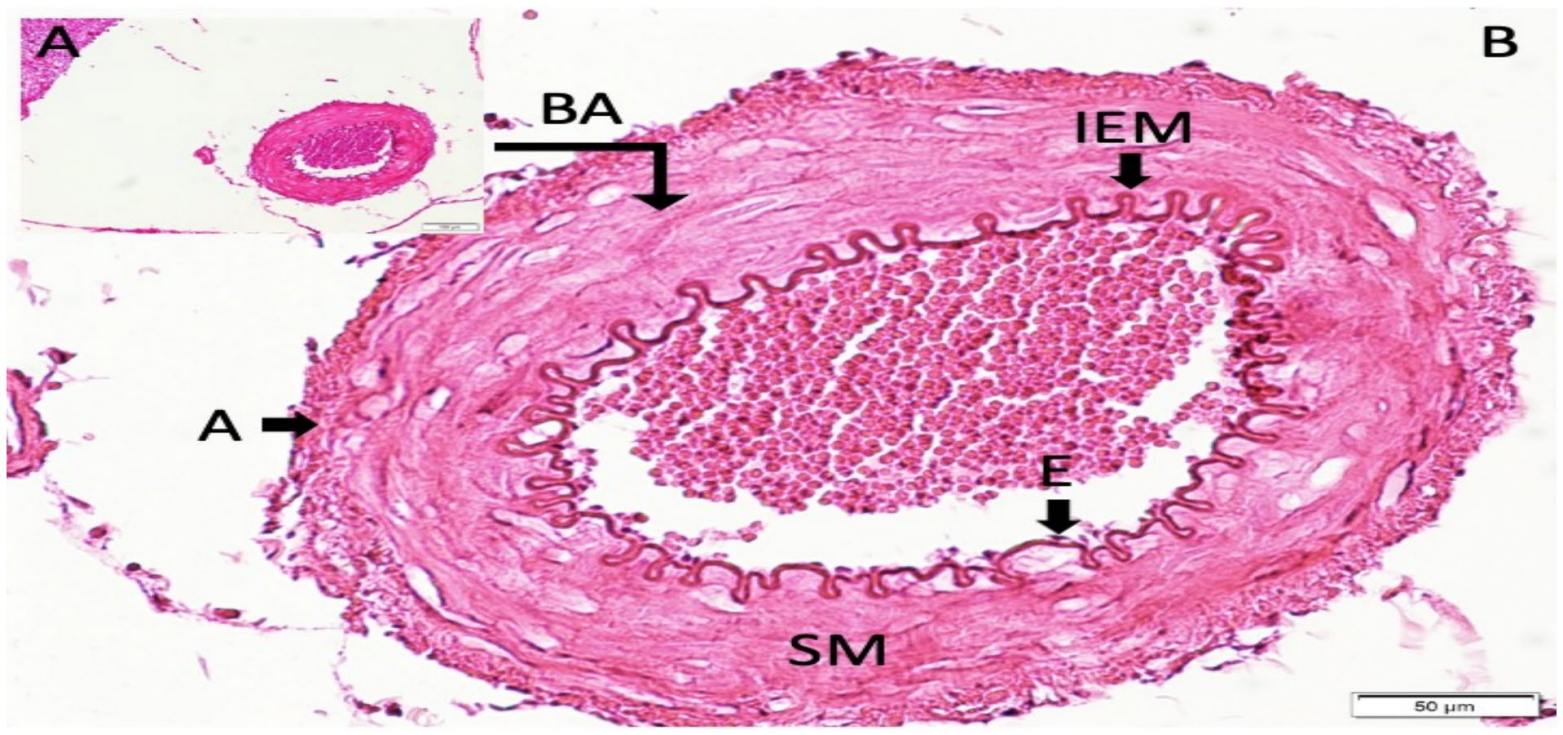
Histopathological appearances of basilar artery (BA) (LM, H&E, x10/A); moderately constructed smooth muscles (SM), moderately injured endothelial cells (E), moderately convoluted inner elastic membrane (IEM) and minimally thickened adventitia (A) (LM, H&E, 40/B) is seen in a male SHAM rabbit.

**Figure 4:**
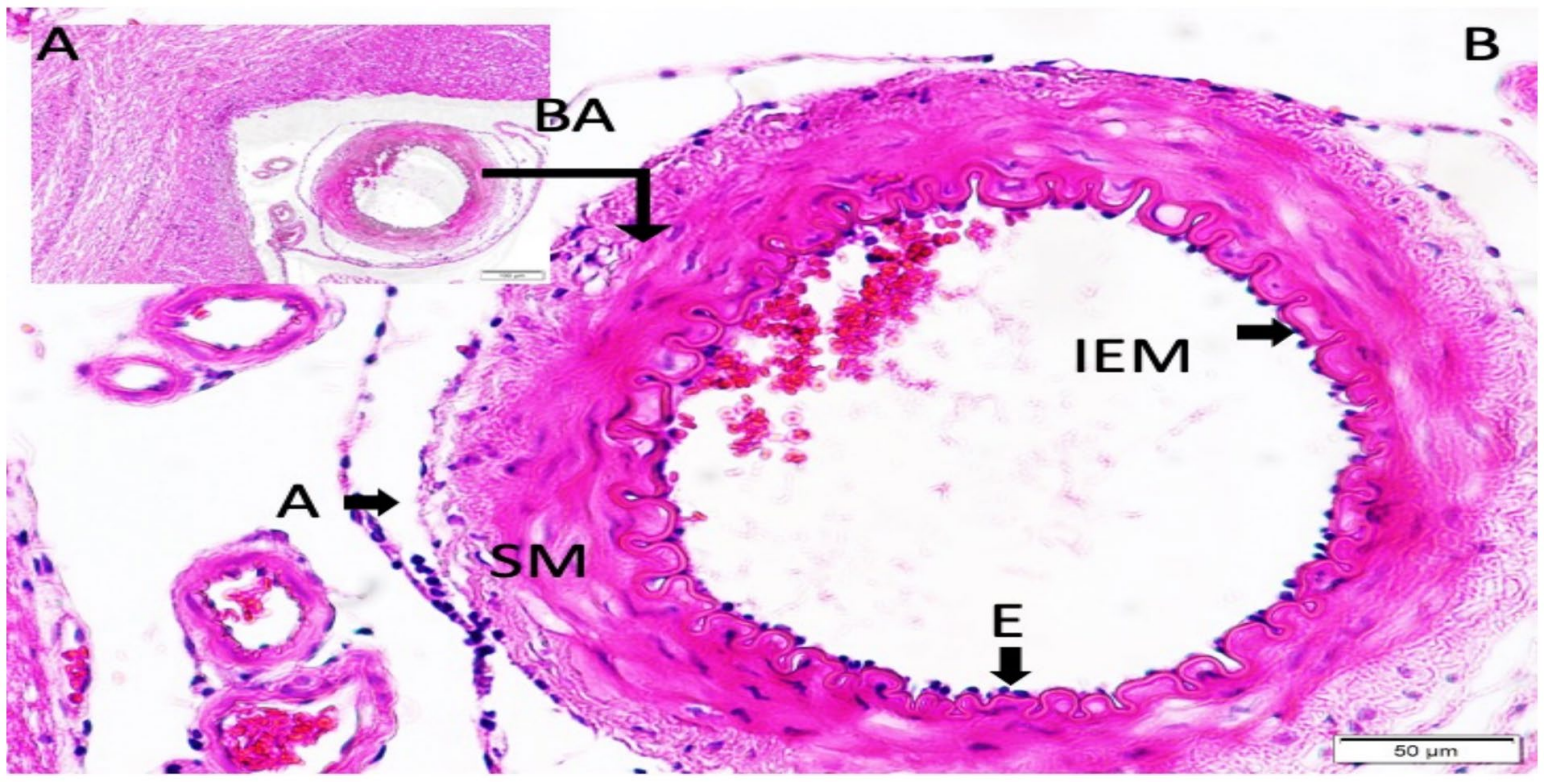
Histopathological appearances of basilar artery (BA) (LM, H&E, ×10/A); minimally constructed smooth muscles (SM), minimally injured endothelial cells (E), minimally convoluted inner elastic membrane (IEM) and thickened adventitia (A) (LM, H&E, 40/B) is seen in a female SHAM rabbit.

SHAM animals exhibited only mild vasospastic changes, consistent with procedural irritation. In male SHAM BAs, we observed moderate SM contraction, minor endothelial injury, and slight IEM convolution (Figure 3). In contrast, female SHAM BAs showed minimal alterations with largely preserved structure (Figure 4).

#### SAH

**Figure 5:**
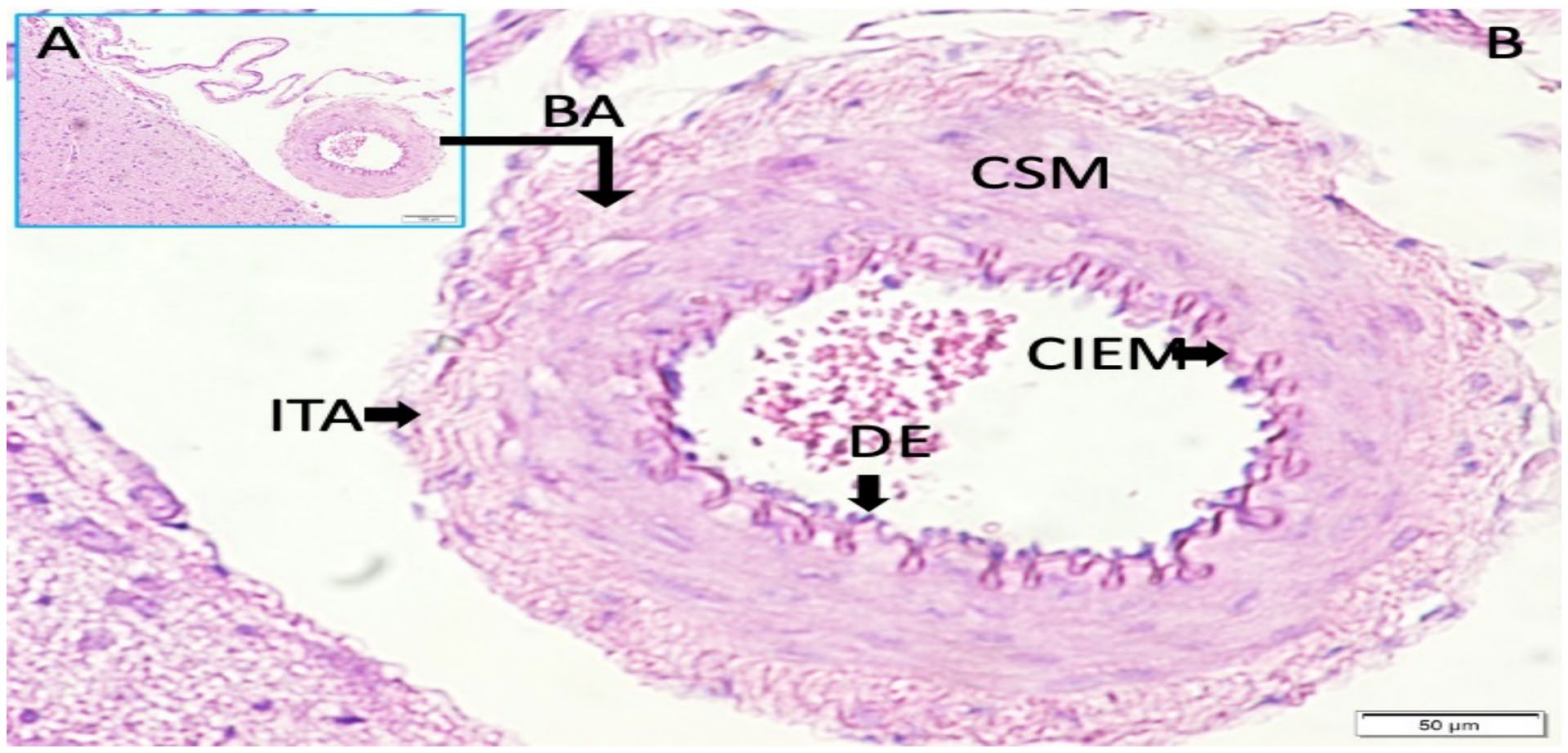
Histopathological appearances of basilar artery (BA) (LM, H&E, ×10/A); severely constructed smooth muscles (SM), severely injured deformed endothelial cells (DE), severely convoluted inner elastic membrane (CIEM) and thickened adventitia (A) (LM, H&E, 40/B) is seen in a male study rabbit.

**Figure 6:**
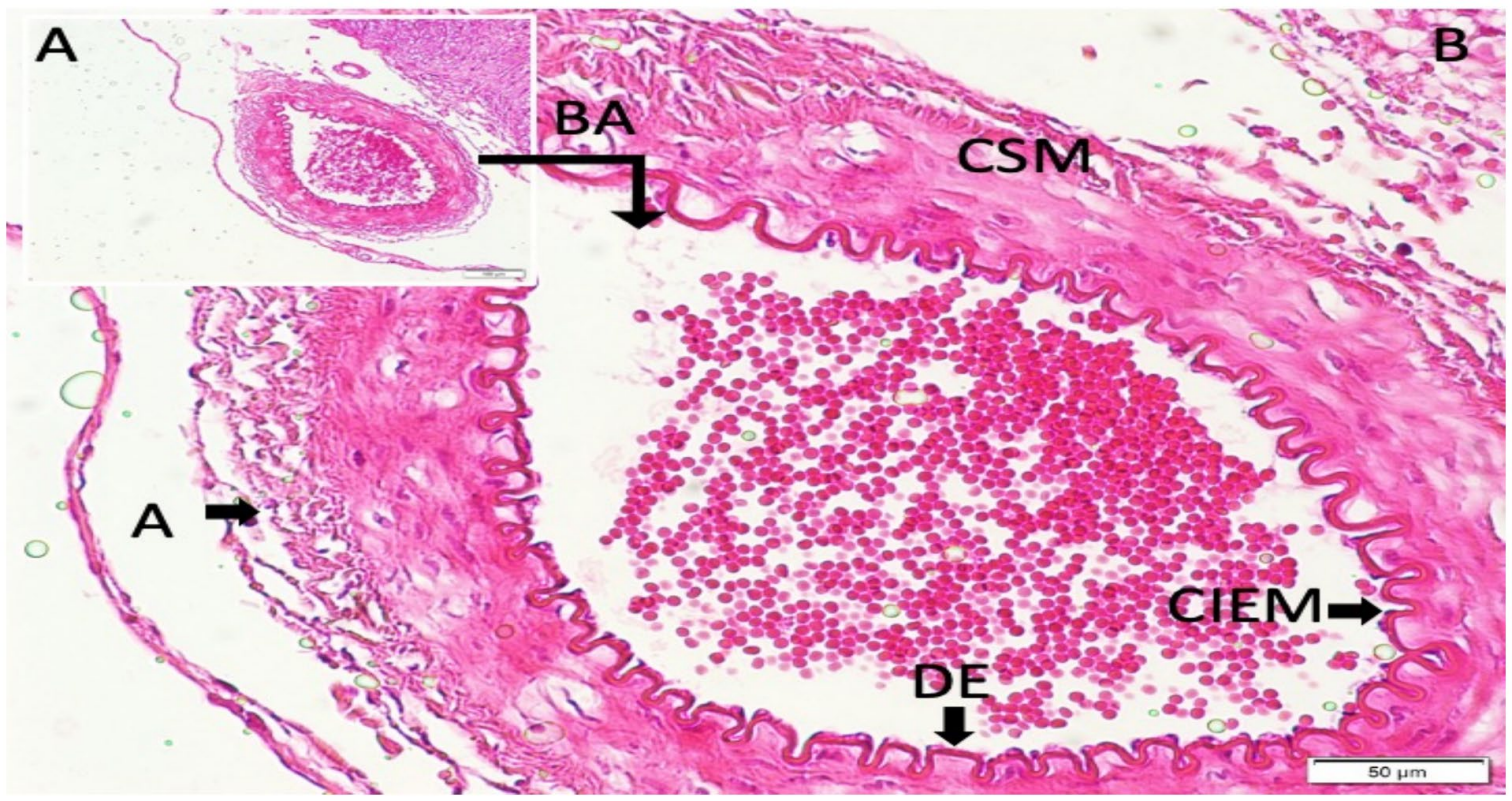
Histopathological appearances of basilar artery (LM, H&E, ×10/A); constructed smooth muscles (SM), injured deformed endothelial cells (DE), convoluted inner elastic membrane (CIEM) and thickened adventitia (A) (LM, H&E, 40/B) is seen in a female study rabbit.

In SAH animals, vasospastic alterations were most striking and clearly sex-dependent. Male SAH BAs exhibited severe SM contraction, markedly deformed/fragmented endothelial cells, a convoluted IEM, and a thickened, inflamed adventitia (Figure 5). Female SAH BAs demonstrated moderate vasospasm, with less pronounced endothelial injury, milder IEM convolution, and moderate adventitial thickening (Figure 6 and see also Supplementary Figure).

### Quantitative assessment of vasospasm

**Figure 7:**
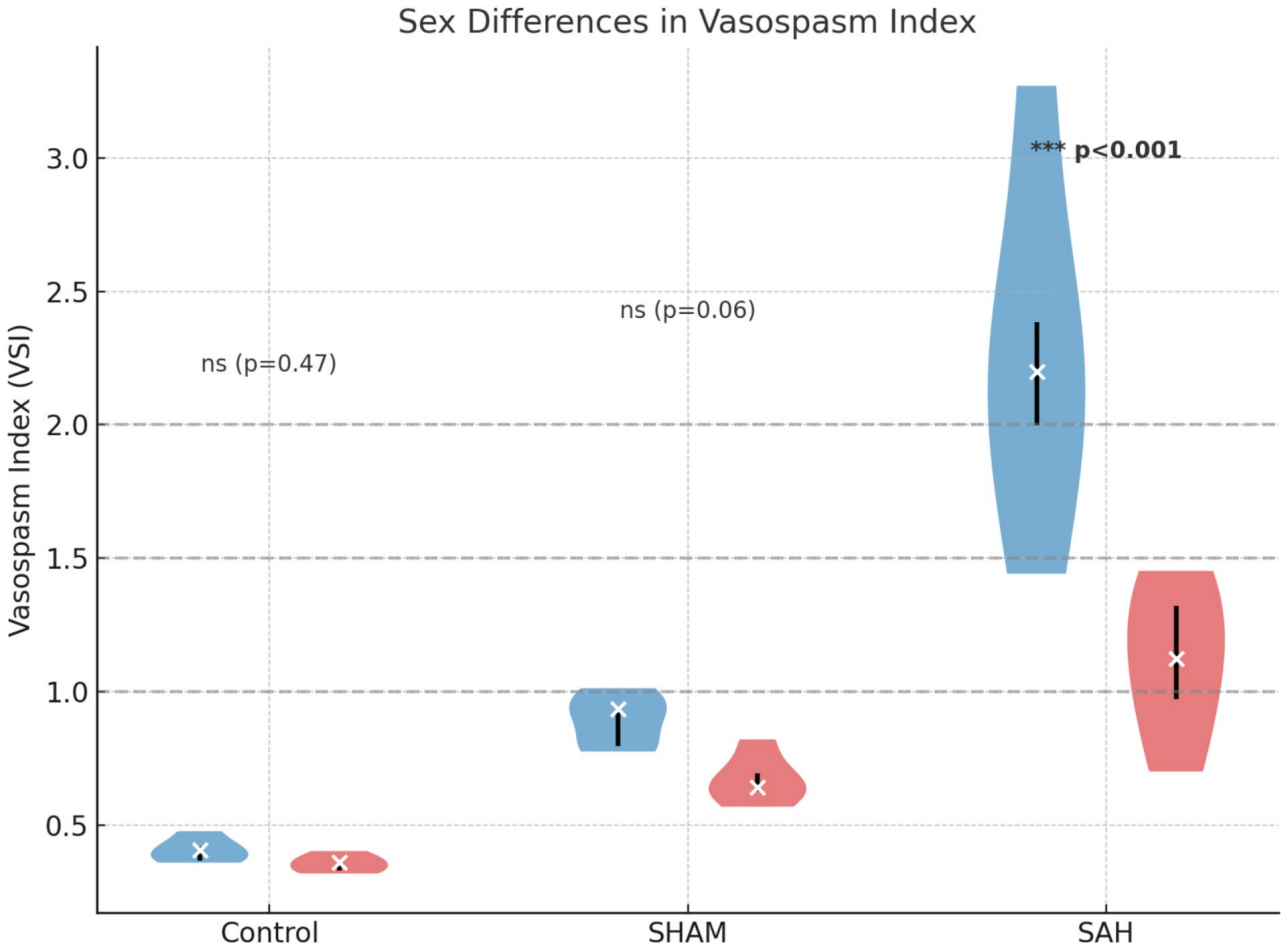
Violin plots of Vasospasm Index (VSI) by sex across Control, SHAM, and SAH. White dots mark medians; boxes denote IQR. *p* values reflect Mann–Whitney *U* (male (Blue) vs. female (Red) within each condition): Control *p* = 0.47; SHAM *p* = 0.06; SAH *p* < 0.001. (Color-coding by sex is used consistently across panels). ***, Statistically strong difference between two groups.

Vasospasm severity was quantified using the Vasospasm Index (VSI). Baseline (Control) values were low and comparable across sexes (male 0.376 ± 0.067 vs. female 0.339 ± 0.041, *p* = 0.47). In SHAM animals, VSI increased modestly, with males (0.987 ± 0.110) showing a trend toward higher values compared with females (0.776 ± 0.146, *p* = 0.06). The most pronounced differences were observed in SAH groups: males exhibited severe vasospasm (2.343 ± 0.632), whereas females showed moderate vasospasm (1.268 ± 0.492); this sex difference was highly significant (*p* < 0.001). The geometric approach used to derive VSI is illustrated in Figure 7, and the summary statistics are presented in Table 1. Consistent with VSI, median histopathology scores were higher in male SAH than female SAH for endothelial injury and IEM convolution, with a similar trend for adventitial thickening (see Supplementary Table S2 for score distributions by feature and group).

**Table 1:** Basilar artery vasospasm index (VSI) in male vs. female rabbits across experimental groups.

Group	Male VSI	Female VSI	Severity (male vs. female)	*p*-Value
Control	0.376 ± 0.067	0.339 ± 0.041	No vasospasm (baseline)	0.47 (ns)
SHAM	0.987 ± 0.110	0.776 ± 0.146	Mild vs. mild	0.06 (ns)^a^
SAH	2.343 ± 0.632	1.268 ± 0.492	Severe vs. moderate	<0.001

Values are mean ± SD. Severity categories: mild (1.0–1.5), moderate (1.5–2.0), severe (>2.0). *p*-Values are from Mann–Whitney *U* tests comparing male vs female within each group.

a Trend toward higher VSI in male SHAM group, not significant at *α* = 0.05. ns = not significant. The median [IQR] VSI values for each group and sex are reported in Supplementary Table S1.

## Discussion

Our results demonstrate that biological sex is a critical factor influencing the severity of vasospasm following subarachnoid hemorrhage (SAH) in this rabbit model. Male rabbits consistently developed more severe basilar artery narrowing and histopathological damage after SAH than female rabbits. This finding is noteworthy when contrasted with clinical observations in humans, where epidemiological studies report that women have a higher incidence of aneurysmal SAH and often worse clinical outcomes than men (5). Although female patients predominate in SAH cohorts and may experience higher rates of complications such as vasospasm and delayed cerebral ischemia in clinical settings, our controlled experimental data showed the opposite pattern: male subjects were more vulnerable to SAH-induced vasospasm than females, as evidenced by a markedly higher Vasospasm Index (VSI; ~2.34 vs. ~1.27). This paradox highlights the complexity of sex-related cerebrovascular responses and suggests that distinct mechanisms may operate across species and biological contexts. A key reconciler is the age–hormone context: our young adult female rabbits likely benefited from high endogenous estrogen levels, whereas aneurysmal SAH in humans peaks in mid-to-late life, when many women are postmenopausal. Estrogen loss may therefore invert the relative risk profile observed in younger females, aligning our preclinical findings with the increased clinical vulnerability of older women (5, 12, 13).

Several biological factors may explain the more severe vasospasm observed in male rabbits. One major consideration is the role of sex hormones in endothelial function. Estrogen and progesterone are well recognized for their vasculoprotective effects, with estrogen promoting endothelial nitric oxide (NO) production, preserving endothelial nitric oxide synthase (eNOS) activity, suppressing inducible NOS (iNOS), and reducing endothelin-1 expression, thereby favoring vasodilation and vascular repair (14–16). Experimental SAH studies in rodents demonstrate that administration of 17β-estradiol (E2) significantly attenuates cerebral vasospasm through preservation of eNOS, suppression of iNOS, and reduction of endothelin-1 (8, 17). Consistent with this evidence, female rabbits in our study exhibited significantly lower VSI values, whereas males—who have minimal estrogen-mediated vascular protection—may experience uninhibited vasoconstriction and heightened oxidative endothelial injury following SAH (18, 19).

Inherent structural and receptor-level differences may further contribute. Males often exhibit greater adrenergic vasoconstrictor receptor density or sensitivity in vascular smooth muscle, and experimental data indicate higher endothelin-A receptor–mediated vasoconstrictor tone in males than females (20). In addition, sex steroid receptor signaling influences vascular architecture and endothelial integrity across multiple levels (2). We emphasize that hormone levels and NO production were not directly measured in this study; thus, interpretations regarding hormonal modulation remain inferential. Nonetheless, the estrogen–NO hypothesis is well supported by established vascular physiology and experimental evidence (8, 18, 21). *In vitro* studies show that E2 significantly increases NO production and eNOS activation in cerebral endothelial cells via receptor-dependent mechanisms, and animal SAH models demonstrate that E2 administration attenuates vasospasm while preserving eNOS expression and suppressing iNOS and endothelin-1, aligning with our observed sex-specific differences (18, 19, 21).

Another potential contributor to sex-specific vasospasm severity is differential sympathetic nervous system (SNS) activity. Cerebral arteries, including the basilar artery, receive sympathetic innervation from cervical ganglia, and excessive sympathetic stimulation has been implicated in vasospasm. Experimental degeneration of the superior cervical sympathetic ganglia reduces basilar artery vasospasm after SAH, suggesting that sympathetic drive exacerbates vasoconstriction (22). Sympathetic hyperactivity has also been shown to adversely affect vascular tone in SAH and may be mitigated by sympathetic blockade in experimental and clinical settings (23). If male rabbits possess a higher baseline sympathetic tone or a more reactive SNS response to stress, as suggested by broader physiological data, this could amplify post-hemorrhagic vasoconstriction and worsen vasospasm (24, 25). Although sympathetic activity was not directly measured in this study, experimental SAH data indicate that post-hemorrhagic catecholamine surges are temporally associated with vasospasm development (24–26).

Beyond hormonal and autonomic influences, sex differences in neuroinflammation may also shape cerebrovascular outcomes. In our study, both male and female SAH rabbits demonstrated classic features of SAH pathophysiology, including early brain injury characterized by increased intracranial pressure, reduced cerebral perfusion, and robust inflammatory activation of the vessel wall and meninges. This aligns with established evidence that SAH elicits a systemic inflammatory response (clinically manifesting as fever and leukocytosis) and that inflammation is strongly associated with vasospasm and poor outcomes (27, 28). We observed adventitial leukocyte infiltration and degenerative changes such as endothelial and smooth muscle cell disruption in all SAH animals, reinforcing the central role of inflammation in SAH pathology. Experimental models further demonstrate that inflammation contributes not only to vasospasm but also to early brain injury and delayed neurological decline (29).

Recent therapeutic advances suggest that targeting inflammatory mediators and toxic blood components can attenuate both early brain injury and vasospasm after SAH. Beyond structural vascular narrowing, accumulating evidence indicates that vasospasm represents an inflammation-driven cerebrovascular process. In particular, interleukin-6 (IL-6) has emerged as a key mediator linking subarachnoid blood exposure to delayed vasospasm and subsequent delayed cerebral ischemia, with human data demonstrating predictable cerebrospinal fluid IL-6 surges preceding vasospasm onset and blood–brain barrier disruption (30). This inflammatory cascade is closely associated with endothelial dysfunction, endothelin-1 activation, microglial signaling, and apoptotic pathways that amplify vascular injury and secondary ischemia. In parallel, hemoglobin-mediated oxidative toxicity further potentiates inflammation; accordingly, hemoglobin scavenging strategies, such as the use of haptoglobin concentrates, reduce hemoglobin-induced inflammatory injury and have shown promise in experimental SAH models (31–33). Thus, while inflammation represents a universal component of SAH pathophysiology across both sexes, sex-dependent differences in inflammatory magnitude, timing, or clearance (potentially involving cytokine signaling and microglial responses) may critically influence vasospasm severity and treatment efficacy. Anti-inflammatory and blood-scavenging interventions may therefore benefit both males and females, although their therapeutic impact is likely to vary by sex-specific inflammatory biology.

Collectively, our findings reinforce a growing recognition that sex is a fundamental biological variable shaping cerebrovascular responses to injury. Accumulating evidence indicates that sex-biased gene expression signatures and sex-steroid receptor signaling may influence endothelial and vascular smooth muscle behavior after hemorrhagic insults, thereby modulating inflammatory, oxidative, and angiogenic pathways (34, 35). Recent vascular research highlights substantial sex-specific differences in endothelial cell function, particularly in inflammatory and oxidative stress responses, while transcriptomic studies demonstrate persistent sex-biased gene expression patterns that affect vascular disease susceptibility from birth into adulthood (36–38). In addition to hormone-dependent mechanisms, emerging data suggest that sex chromosome effects (XX versus XY) may represent an additional mechanistic dimension capable of influencing vascular biology independently of circulating hormones (39). Within this framework, our study provides direct preclinical evidence supporting sex-differentiated responses to cerebrovascular injury after SAH, positioning genetic and transcriptomic factors as plausible explanatory layers and important priorities for future mechanistic investigation.

The discrepancy between our experimental findings and clinical outcome trends warrants careful consideration and is plausibly explained, at least in part, by age- and hormone-dependent biological context. Our animals were young adults, with female rabbits likely benefiting from higher endogenous estrogen levels, whereas aneurysmal SAH in humans disproportionately affects older populations, in which many female patients are postmenopausal. This age-dependent hormonal milieu may shift the direction or magnitude of sex effects observed clinically versus experimentally. Moreover, because our study is a controlled rabbit model focused on anatomical basilar artery vasospasm, effect sizes and risk profiles cannot be directly extrapolated to specific human subpopulations. Differences in age and hormonal status, species-specific cerebrovascular anatomy, and variations in outcome definitions are all likely contributors to the divergence between experimental and clinical data.

Whereas our primary endpoint was anatomical basilar artery vasospasm, clinical studies emphasize outcomes such as DCI, infarction, and functional status, which do not always correlate directly with angiographic vasospasm (40–42). Although a clear association between cerebral vasospasm and DCI exists, accumulating evidence indicates that DCI can involve brain regions remote from sites of large-vessel narrowing and may be driven by additional secondary injury mechanisms. Recent translational work has highlighted the roles of endothelial dysfunction, inflammatory signaling, impaired microcirculatory regulation, and emerging processes such as glymphatic system disruption in the pathophysiology of DCI (43). Accordingly, interventions that effectively reduce angiographic vasospasm do not invariably translate into improved neurological outcomes, underscoring a persistent disconnect between vascular narrowing and tissue-level ischemic injury.

Our findings should be interpreted with appropriate caution. Because our study focused on anatomical vasospasm in a controlled rabbit model, we did not measure DCI, cerebral blood flow, or functional neurological outcomes and therefore cannot infer sex-specific DCI risk from our dataset. Moreover, human SAH risk, vasospasm and DCI susceptibility, and overall outcomes are shaped by substantial population-level heterogeneity, including age distribution, comorbidities, aneurysm characteristics, and complication profiles. Worse outcomes observed in women clinically may thus reflect factors beyond vasospasm alone such as higher rates of DCI and hydrocephalus rather than a simple inversion of the vascular response observed in young experimental animals (5, 6, 44). While key pathophysiological pathways implicated in vasospasm, including endothelial dysfunction, inflammation, and autonomic influences, are biologically conserved across species, differences in cerebrovascular biology and vasospasm evolution constrain direct translation. Together, these considerations highlight the need for future studies incorporating cerebral blood flow measures, DCI-aligned endpoints, and age- and hormone-stratified designs to better define sex-specific mechanisms linking vasospasm to delayed ischemic injury.

Several limitations should be acknowledged. Although the rabbit model enables controlled experimentation, it differs from humans in cerebrovascular anatomy and life history. Subgroup sample sizes were modest, estrous cycle stage and circulating hormone levels were not recorded, and mechanistic biomarkers related to endothelial function, inflammation, and sympathetic activity were not measured. Our analysis focused on anatomical vasospasm of the basilar artery without assessing functional neurological outcomes, multi-territorial vascular involvement, or cerebral blood flow. In addition, the sham procedure induced mild vasospastic changes, indicating that surgical manipulation itself can contribute to vascular responses. Nevertheless, the differences between SAH and sham groups, and between male and female SAH animals, were sufficiently large to support the robustness of our conclusions.

Despite these limitations, our study provides important insight into how sex differences influence cerebrovascular outcomes after hemorrhagic stroke. The pronounced disparity in vasospasm severity between male and female rabbits suggests that inherent biological factors tied to sex (including hormonal milieu, immune responses, and vascular signaling) modulate SAH-induced vasculopathy. These findings challenge a one-size-fits-all paradigm and underscore the importance of incorporating sex as a biological variable in experimental design and translational interpretation. Future studies integrating hormonal, inflammatory, autonomic, and genomic analyses will be essential to elucidate the mechanisms underlying these differences and to guide sex-aware therapeutic strategies.

## Conclusion

In this experimental rabbit model of subarachnoid hemorrhage, male subjects consistently exhibited more severe vasospastic changes in the basilar artery than female subjects. Histopathologically, male SAH rabbits demonstrated classic hallmarks of severe vasospasm, including a markedly convoluted internal elastic membrane, significant luminal narrowing with deformed endothelium, hypercontracted smooth muscle layers, and a thickened, inflamed adventitia, whereas female SAH rabbits showed only moderate alterations. These findings provide clear evidence that sex plays a pivotal role in determining cerebrovascular injury severity after SAH. Recognizing this sex difference challenges a one-size-fits-all approach and supports the development of sex-specific therapeutic strategies. Incorporating sex into both research design and clinical management may ultimately enable more personalized and effective interventions for patients suffering from this devastating condition.

## Data Availability

The raw data supporting the conclusions of this article will be made available by the authors, without undue reservation.
