# Supplementary material for: Sex-specific differences in basilar artery vasospasm after subarachnoid hemorrhage: evidence from a rabbit model
Source: Front Neurol. 2026 Jun 3;17:1739644. doi: 10.3389/fneur.2026.1739644 (PMC13271970; doi:10.3389/fneur.2026.1739644)
Supplement: Supplementary file 1 [file Supplementary_File_1.DOCX]

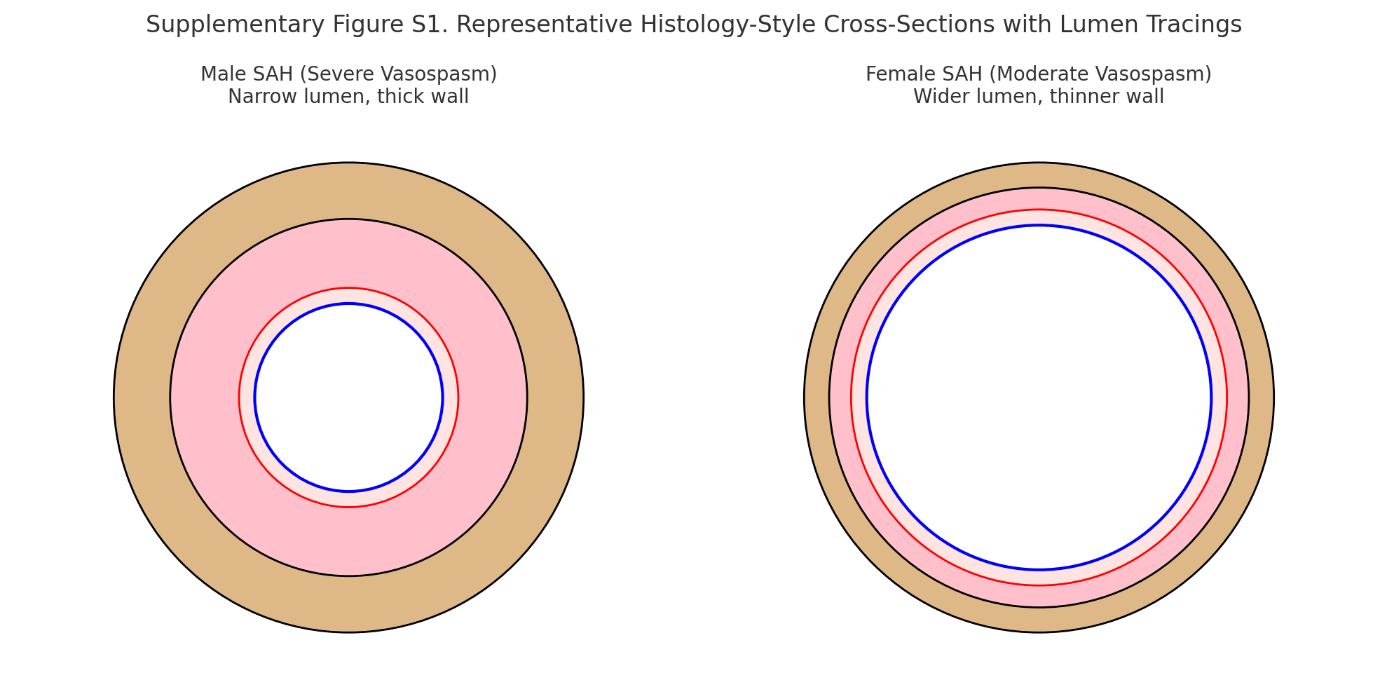


• **Male SAH (left):** lumen is very narrow, wall is thick, layered adventitia/media/endothelium are exaggerated → severe vasospasm.

• **Female SAH (right):** lumen is larger, wall thinner, overall architecture less constricted → moderate vasospasm.

• Color-coding simulates H&E histology layers:

- **Adventitia** (light brown),
- **Media** (pink),
- **Endothelium** (red boundary),
- **Lumen** (white with blue tracing).
