# Supplementary material for: Sex-specific differences in basilar artery vasospasm after subarachnoid hemorrhage: evidence from a rabbit model
Source: Front Neurol. 2026 Jun 3;17:1739644. doi: 10.3389/fneur.2026.1739644 (PMC13271970; doi:10.3389/fneur.2026.1739644)
Supplement: Supplementary file 2 [file Table_1.DOCX]

Supplementary Table S1: Basilar Artery Vasospasm Index (VSI)

Title: Supplementary Table S1. VSI values by sex and condition (median [IQR] and mean ± SD)

| **Group** | **Male VSI (median [IQR])** | **Male VSI (mean ± SD)** | **Female VSI (median [IQR])** | **Female VSI (mean ± SD)** |
| --- | --- | --- | --- | --- |
| Control | 0.38 [0.34–0.41] | 0.376 ± 0.067 | 0.34 [0.32–0.36] | 0.339 ± 0.041 |
| SHAM | 0.99 [0.91–1.05] | 0.987 ± 0.110 | 0.78 [0.69–0.88] | 0.776 ± 0.146 |
| SAH | 2.30 [1.87–2.76] | 2.343 ± 0.632 | 1.22 [0.89–1.58] | 1.268 ± 0.492 |
