# Supplementary material for: Sex-specific differences in basilar artery vasospasm after subarachnoid hemorrhage: evidence from a rabbit model
Source: Front Neurol. 2026 Jun 3;17:1739644. doi: 10.3389/fneur.2026.1739644 (PMC13271970; doi:10.3389/fneur.2026.1739644)
Supplement: Supplementary file 3 [file Table_2.DOCX]

Supplementary Table S2: Ordinal Histopathology Scores

Title: Supplementary Table S2. Distribution of histopathology scores (median [IQR]) by sex and condition

| **Group** | **Smooth Muscle Contraction** | **Endothelial Integrity** | **IEM Configuration** | **Adventitial Thickness** |
| --- | --- | --- | --- | --- |
| Control – Male | None (0 [0–0]) | None (0 [0–0]) | Normal (0 [0–0]) | None (0 [0–0]) |
| Control – Female | None (0 [0–0]) | None (0 [0–0]) | Normal (0 [0–0]) | None (0 [0–0]) |
| SHAM – Male | Mild–Moderate (2 [1–2]) | Mild (1 [1–2]) | Mild (1 [1–2]) | Mild (1 [0–1]) |
| SHAM – Female | Mild (1 [0–1]) | Minimal (0–1) | Minimal (0–1) | Mild (1 [1–2]) |
| SAH – Male | Severe (3 [3–4]) | Severe (3 [3–4]) | Severe (3 [3–4]) | Severe (3 [2–3]) |
| SAH – Female | Moderate (2 [2–3]) | Moderate (2 [2–3]) | Moderate (2 [2–3]) | Moderate (2 [2–3]) |
